# TiNi-Based Bi-Metallic Shape-Memory Alloy by Laser-Directed Energy Deposition

**DOI:** 10.3390/ma15113945

**Published:** 2022-06-01

**Authors:** Yitao Chen, Cesar Ortiz Rios, Braden McLain, Joseph W. Newkirk, Frank Liou

**Affiliations:** 1 Department of Mechanical and Aerospace Engineering, Missouri University of Science and Technology, Rolla, MO 65409, USA; btmywv@mst.edu (B.M.); liou@mst.edu (F.L.); 2 Department of Materials Science and Engineering, Missouri University of Science and Technology, Rolla, MO 65409, USA; codqc@mst.edu (C.O.R.); jnewkirk@mst.edu (J.W.N.)

**Keywords:** additive manufacturing, directed energy deposition, shape-memory alloys, joining of metals, elemental powders

## Abstract

In this study, laser-directed energy deposition was applied to build a Ti-rich ternary Ti–Ni–Cu shape-memory alloy onto a TiNi shape-memory alloy substrate to realize the joining of the multifunctional bi-metallic shape-memory alloy structure. The cost-effective Ti, Ni, and Cu elemental powder blend was used for raw materials. Various material characterization approaches were applied to reveal different material properties in two sections. The as-fabricated Ti–Ni–Cu alloy microstructure has the TiNi phase as the matrix with Ti_2_Ni secondary precipitates. The hardness shows no high values indicating that the major phase is not hard intermetallics. A bonding strength of 569.1 MPa was obtained by tensile testing, and digital image correlation reveals the different tensile responses of the two sections. Differential scanning calorimetry was used to measure the phase-transformation temperatures. The austenite finishing temperature of higher than 80 °C was measured for the Ti–Ni–Cu alloy section. For the TiNi substrate, the austenite finishing temperature was tested to be near 47 °C at the bottom and around 22 °C at the upper substrate region, which is due to the repeated laser scanning that acts as annealing on the substrate. Finally, the multiple shape-memory effect of two shape-memory alloy sides was tested and identified.

## 1. Introduction

Joining different metal alloys into a single bi-metallic component can realize a wide variety of combinations with excellent material properties [1,2,3], and the joining of bi-metals is necessary in many industrial environments where different properties are needed at different locations [3]. The directed energy deposition (DED) additive manufacturing (AM) process has become an important approach to realize various types of metal joining and metal repair since strong metallurgical bonding can be achieved [4,5,6]. So far, DED has been used in various applications including metal joining and part repairing [2,6,7,8]. Also, since the AM process has a high degree of freedom for the spatial distribution of both geometry and material compositions, it can be much more flexible in developing more novel alloy structures [9,10]. Typical works using DED to join similar or dissimilar alloys include steels, Ti alloys, Ni-based superalloys, and Cu alloys. Sahasrabudhe et al. [2] joined SS410 with Ti-6Al-4V to obtain a high application temperature at the Ti-alloy end and the economic corrosion-resistive steel end. Jones et al. [11] applied DED for the manufacturing of steel-Inconel bi-metallic structures. Zhang et al. [12] used DED to join steels and Cu in order to obtain both high strengths from steel and high thermal conductivity from Cu. Functionally graded structures and interlayers can also be used for joining two dissimilar alloy parts by DED, which benefits from the flexible nature of the additive process [13,14,15].

Shape-memory alloy (SMA) is a type of smart metallic material that is able to demonstrate different shapes at different temperatures and memorize its original shape through austenite-martensite phase transformation [16,17]. TiNi alloy is a popular metallic material that can demonstrate shape-memory effects [17,18,19]. For TiNi alloys, it has been found that their phase-transformation temperature (TT) can be changed by adjusting the chemical composition and processing methods [17,20]. Therefore, the TTs of TiNi alloy can cover a wide temperature range for multiple functions [20]. Also, if different types of TiNi SMAs can coexist in a single component, it will obtain more unique and attractive multiple shape-memory behaviors. For example, Khan et al. [21] flexibly applied a laser to TiNi alloys to generate a discrepancy between laser-affected areas and created location-dependent TTs within a single part. Nematollahi et al. [22] applied selective laser melting (SLM), a powder-bed-based AM process, to build a graded TiNi SMA by changing the processing parameters at two different sections. For DED, by taking advantage of the flexible capability in similar or dissimilar joining, SMAs with multiple shape-memory behaviors can also be combined using DED to demonstrate more functions. Most of the aforementioned DED processes used for metal joining focused on improving the comprehensive mechanical, structural, and thermal properties. However, the joining effect and shape-memory behavior after joining two different types of TiNi-based SMAs using DED have not been widely reported.

For DED, both the feedstock SMA materials and the substrate SMA materials can have a wide range of selections. In this paper, we applied the DED approach to join two different TiNi-based parts into a bi-metallic SMA in order to exhibit multiple shape-memory behaviors. A Ti-rich ternary Ti–Ni–Cu SMA was deposited on a near equiatomic binary TiNi alloy to realize the bi-metallic SMAs, which were joined by two SMA parts with different element types, element compositions, and processing methods. A small amount of a third element can be applied to create more applications [23], such as by adding Cu to replace a small portion of Ni, Ti–Ni–Cu SMA can obtain narrower hysteresis and potential applications in the biomedical field [24,25]. The Ti–Ni–Cu ternary SMA was fabricated by the powder mixture of Ti, Ni, and Cu elemental powders. The elemental powder mixture was used as an alternative form of raw materials other than pre-alloyed powders [26,27]. The SMA with an as-mixed atomic composition of Ti–45at.%Ni–5at.%Cu was fabricated by elemental powders in [28,29] with titanium as the substrate material. In this work, more details will be studied on using elemental powder DED to build Ti–Ni–Cu/TiNi bi-metallic alloys. The joining effect at the interface, the bonding strength, and the multiple functional behaviors from different sections of SMA will all be evaluated.

## 2. Materials and Methods

**Figure 1:**
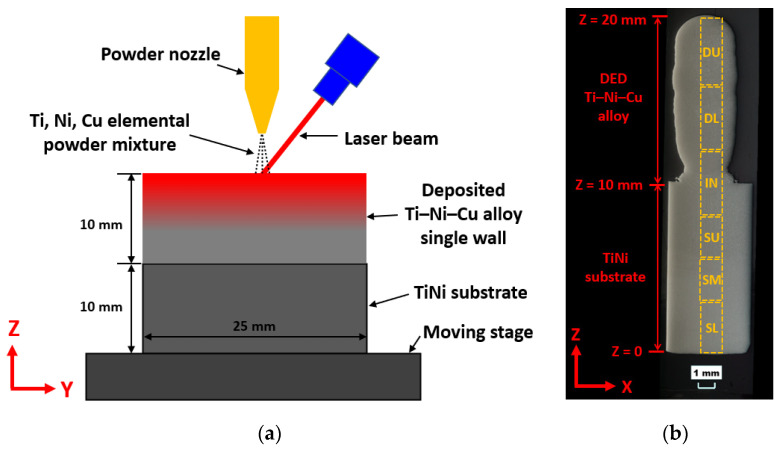
(**a**) The schematic of joining bi-metallic SMA using DED in this work. (**b**) Image of the XZ-plane cross-section of the bi-metallic structure including the TiNi substrate and the DED Ti–Ni–Cu alloy. The bottom line of the substrate has been marked as Z = 0. The Z height of the interface and the top point are 10 mm and 20 mm, respectively.

In this work, a Ti-rich Ti–Ni–Cu alloy single-wall structure was deposited on a TiNi substrate by a DED processing system mainly including a laser system, a CNC-controlled moving stage, and a powder feeder. Near spherical Cp-Ti powder (AP&C, particle size 53~150 μm), Ni powder (Atlantic Equipment Engineers Inc., Upper Saddle River, NJ, USA, particle size −100/+325 mesh), and Cu powder (Royal Metal Powders Inc., Maryville, TN, USA, average particle size 110 μm [12]) were mixed with the atomic composition of Ti/Ni/Cu = 53/44/3 to create a Ti-rich composition that is different from the TiNi substrate. We use Ti–Ni–Cu alloy to represent this Ti–44at.%Ni–3at.%Cu ternary SMA that was deposited and studied in this paper. The powder blend was homogenized using a Turbula T2F mixer for 0.5 hr. The substrate material is near-equiatomic TiNi binary SMA bar material purchased from Kellogg’s Research Labs (New Boston, NH, USA), which was cut to size, 25 mm × 10 mm × 5 mm. The Ti–Ni–Cu alloy single-wall deposition strategy on the TiNi substrate is illustrated in Figure 1a. The substrate was fixed by a vise on the moving stage, and the single wall structure was deposited on the surface of 25 mm × 5 mm of the substrate. An IPG Photonics CW fiber laser with a 1064 nm wavelength was used in this work to create a melt pool on the substrate. The spot size was adjusted and fixed at approximately 2.5 mm. A powder nozzle was used to feed powders into the melt pool and the nozzle was 10 mm above the substrate surface. The moving stage followed a single track and multilayer toolpath. The toolpath consists of uniform +Y direction and −Y direction movements with no dwell time for multiple cycles. Between each change in the Y direction, the stage dropped down vertically by one increment, which represents the layer thickness. The travel speed for the stage in the +Y and −Y directions was 250 mm/min. During the traveling guided by the toolpath, the Ti, Ni, and Cu powder mixture was delivered into the melt pool through the powder nozzle by the powder feeder supplied by Powder Motion Labs. Solid layers build up along the Z direction as seen in Figure 1a,b, which is called the building direction (BD). The laser power was set at 600 W at the first layer and 400 W for all the remaining layers. A total of 60 layers were deposited, and the dimension of the DED Ti–Ni–Cu alloy along the Y direction (long direction) is 25 mm, whereas the total deposition height of the Ti–Ni–Cu alloy is 10 mm. The entire process was carried out within an argon atmosphere to minimize the oxidation effect. 

The cross-section of the 10 mm tall Ti–Ni–Cu alloy single wall joined with the 10 mm tall TiNi substrate was cut off by wire-EDM and was prepared for material characterization, including microstructural, functional, and mechanical testing and joining evaluation. A sample from the cross-section along the XZ-plane of the bi-metallic structure was prepared by following the metallographic specimen preparation steps using a Buehler EcoMet 250 grinder-polisher in order to observe the microstructure through microscopes. The sample was mounted by epoxy and ground using SiC sandpapers from 320 grit to 1200 grit. After grinding, the sample was then polished by 9 µm, 6 µm, 3 µm, 1 µm diamond suspension, and 0.05 µm colloidal alumina. After polishing, the sample was etched using Kroll’s reagent (2 mL HF, 6 mL HNO_3_, and 92 mL distilled water) by swabbing the surface for 30 s. Optical microscopic imaging was done on the sample by a Hirox KH-8700 optical microscope (OM) (Hirox Co., Ltd., Tokyo, Japan). High-resolution electron microscopic imaging and energy-dispersive spectroscopy (EDS) analysis were performed by a Thermo Fisher Scientific Helios Hydra CX scanning electron microscope (SEM) (Thermo Fisher Scientific, Waltham, MA, USA) and a Helios NanoLab 600 SEM (Thermo Fisher Scientific, Waltham, MA, USA).

Figure 1b is an OM image of the well-polished cross-section of the bi-metallic SMA structure. To determine the locations, the bottom point of the TiNi substrate is marked as Z = 0. Correspondingly, the interfacial line and the top point of the DED Ti–Ni–Cu alloy single wall are marked as Z = 10 mm and Z = 20 mm, respectively. Vickers hardness values of both the DED Ti–Ni–Cu alloy part and the TiNi substrate were measured by a Struers Duramin-5 Vickers hardness tester. According to the Z coordinate shown in Figure 1b, Vickers hardness testing was conducted at a height from Z = 1 mm to Z = 19 mm with a 1 mm interval. For each height, three indentations were taken, and the average hardness of each height was calculated. Each indentation used a load of 1.96 N and a dwell time of 10 s.

**Figure 2:**
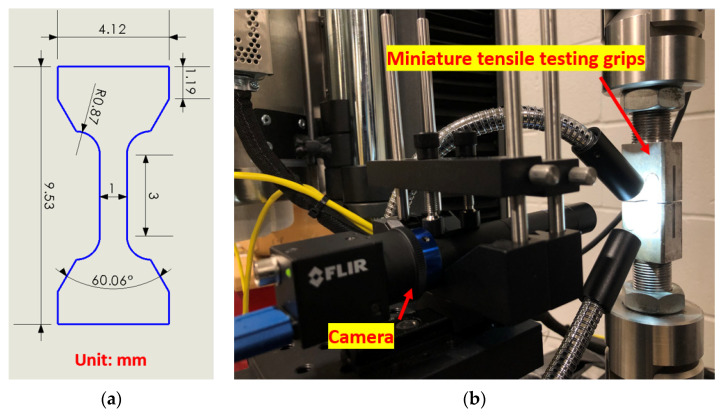
(**a**) The sketch of the miniature tensile specimen design. (**b**) The camera setup for DIC.

The bonding strength between the Ti–Ni–Cu alloy part and the TiNi substrate was measured by uniaxial tensile testing via an Instron 5969 universal testing machine (Instron, Norwood, MA, USA) and analyzed by the digital image correlation (DIC) technique. Figure 2a shows the sketch of the miniature tensile specimen that was developed to study mechanical behaviors of small size materials. The gauge length of the specimen is 3 mm, and the thickness is 1 mm. More information can be found in a previous study [30]. A 10 kN load cell was applied for the testing of the miniature tensile specimen. The crosshead speed was controlled to maintain the strain rate at 0.003 mm/mm/s during the tensile test. Figure 2b displays the camera setup for DIC during the tensile testing. The following fracture surface analysis was also conducted using the Helios Hydra CX SEM.

Differential scanning calorimetry (DSC) was used for studying the TTs of various sections of the as-deposited Ti–Ni–Cu/TiNi bi-metallic structure using a TA Instruments Q2000 differential scanning calorimeter (New Castle, DE, USA) with a ramping heating/cooling rate of 10 °C/min. Apart from DSC, the shape-memory effect of the as-deposited bi-metallic SMA was tested by recording the shape recovery on a hot plate with changing temperatures.

## 3. Results and Discussions

### 3.1. Microstructural Characterization

**Figure 3:**
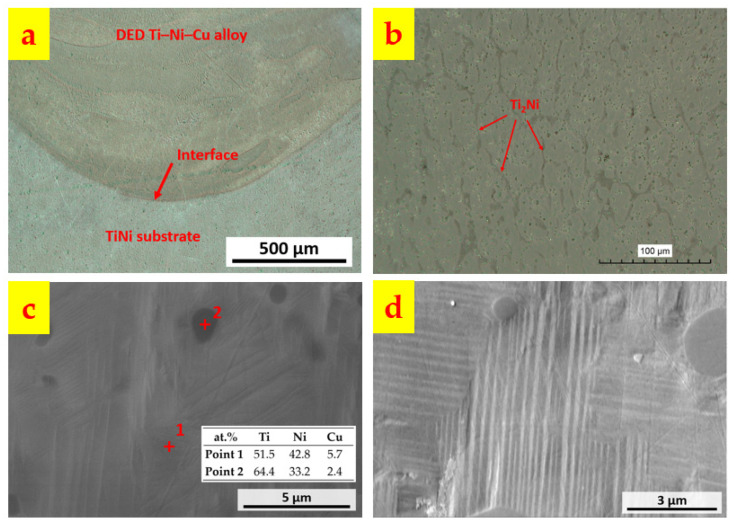
Microstructural features of the as-deposited Ti–Ni–Cu SMA. (**a**) Interfacial region between the DED Ti–Ni–Cu SMA and the TiNi substrate. (**b**) Microstructure of TiNi matrix and Ti_2_Ni. (**c**) EDS point analysis of TiNi matrix and Ti_2_Ni. (**d**) Twinning structure of martensitic TiNi phase.

**Figure 4:**
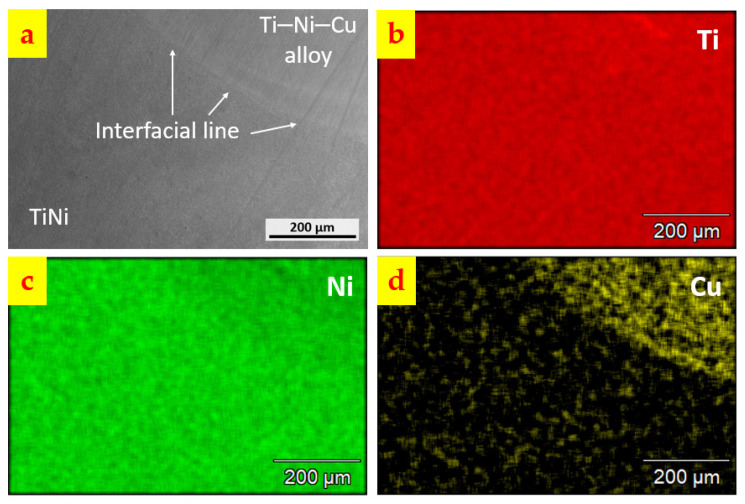
(**a**) Interface between the DED Ti–Ni–Cu alloy and the TiNi substrate. (**b**) Element mapping of Ti. (**c**) Element mapping of Ni. (**d**) Element mapping of Cu. Notice that the intensity of the Cu signal from the Ti–Ni–Cu alloy above the interfacial line is much stronger than the area within the TiNi substrate below the interfacial line.

Figure 3 shows the micrographs of the as-deposited Ti–Ni–Cu alloy. Figure 3a is an OM image of the interface between the DED Ti–Ni–Cu alloy and the TiNi substrate. A clear interfacial line can be observed and no gas pores and cracks are found near the interfacial line, which indicates a good interaction between the powder flow of the first layer and the substrate materials within the melt pool. Figure 3b is obtained from the Ti–Ni–Cu alloy section, where darker phases distribute within the matrix and form a columnar microstructure. With the assistance of SEM/EDS, higher magnification images are acquired and shown in Figure 3c,d. In Figure 3c, EDS points analysis was performed within both the matrix (Point 1) and the dark minor phase (Point 2). The atomic compositions from the EDS analysis of Point 1 and Point 2 are listed in Figure 3c. It can be seen that in the dark phases, the atomic composition of Ti is twice the atomic composition of (Ni + Cu). Therefore, the dark minor phases are Ti_2_Ni intermetallics, which are marked in Figure 3b. The composition of Point 1 indicates that the matrix is Ti-rich TiNi with a Ti atomic composition of about 51.5 at.% and a small number of Cu atoms to replace the Ni atoms. The high magnification image of Figure 3d clearly shows the martensite twinning structure and dark Ti_2_Ni particles. Large-area EDS analysis was also performed at heights of Z = 10 mm, 12 mm, 14 mm, 16 mm, and 18 mm of the Ti–Ni–Cu alloy section. The average atomic composition of the five heights measured by EDS is Ti (52.1 ± 0.5) Ni (43.3 ± 0.9) Cu (4.6 ± 0.4). The fluctuation of Ti, Ni, and Cu compositions among the five heights is much lower than the former work that deposited Ti–45at.%Ni–5at.%Cu alloy on the titanium substrates [29]. The dilution from the TiNi substrate in this work has less influence on the as-deposited section. The SEM image of the interfacial region is demonstrated in Figure 4a. Figure 4b–d are the EDS mapping of the Ti, Ni, and Cu elements. It can be seen that the Cu signal is much weaker below the interfacial line; however, there is not a significant change in the signal of Ti and Ni between the area above and below the interface, since the compositions of Ti and Ni within the Ti–Ni–Cu alloy and TiNi substrate are similar. The Ni signal shows a gradual and slight decrease from the TiNi substrate area to the Ti–Ni–Cu alloy area since the atomic composition of Ni in the substrate is a little higher than the Ti–Ni–Cu alloy.

### 3.2. Hardness Distribution

**Figure 5:**
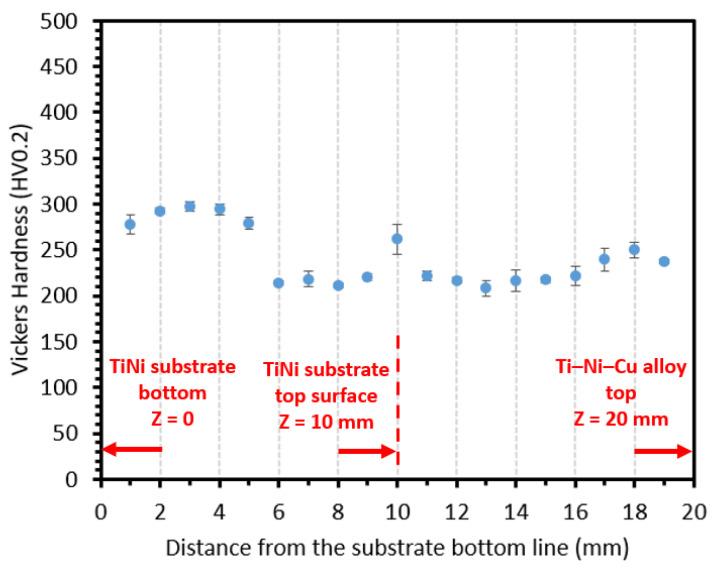
Vickers hardness distribution from the bottom of the TiNi substrate to the top of the DED Ti–Ni–Cu alloy. Notice that the lack of high hardness value from the hard intermetallics indicates that the major phase is the TiNi phase. Relatively higher hardness values can be observed at the lower section of the substrate from Z = 1 mm to Z = 5 mm and at the point of Z = 10 mm near the interface.

Vickers hardness distribution from Z = 1 mm to Z = 19 mm with a 1 mm interval is plotted in Figure 5. Figure 5 shows the average hardness of the three indentations at each Z height. It can be seen that the Vickers hardness distribution from bottom to top shows typical low hardness (200~300 HV0.2) of the entire cross-sectional area along the Z direction of the bi-metallic structure. It was reported in [31] that the hardness of the intermetallic Ti_2_Ni phase could be as high as 700 HV. Thus, the low hardness reflects that the major phase of this bi-metallic structure is the TiNi phase, and the Ti_2_Ni intermetallic phase is the dispersive secondary phase in the as-deposited Ti–Ni–Cu alloy section due to the rich atomic composition of the Ti element. Almost all of the average hardness values of the DED Ti–Ni–Cu alloy (above Z = 10 mm) are within the range of 200~250 HV0.2. Within the substrate, from Z = 1 mm to Z = 9 mm, an obvious difference in the average hardness value can be observed between the lower half of the substrate and the upper half of the substrate. The average hardness values of the lower half of the substrate (Z < 6 mm) are closer to 300 HV0.2. The hardness also obtains a higher value at the location of Z = 10 mm, which could be due to the local mixing of the Ti, Ni, and Cu elements between the TiNi substrate and the first layer of DED Ti–Ni–Cu alloy that might result in a local small composition deviation in the TiNi matrix compared to the original TiNi substrate.

To obtain more information related to the Z-height-dependent hardness value, the sample sectioning plan for the following DSC analysis is designed according to the dashed boxes in Figure 1b. From bottom to top, they are the lower part of the substrate (SL, Z height: 0~3 mm), the middle part of the substrate (SM, Z height: 3~5.5 mm), the upper part of the substrate (SU, Z height: 5.5~8 mm), the interface including both the substrate and the bottom region of the DED Ti–Ni–Cu alloy (IN, Z height: 8~12 mm), the lower part of the DED Ti–Ni–Cu alloy (DL, Z height: 12~16 mm), and the upper part of the DED Ti–Ni–Cu alloy (DU, Z height: 16~20 mm). In this way, the phase-transition behaviors of samples SL and SM represent the lower half region of the substrate with higher average hardness, and sample SU reflects the upper half region of the substrate with lower average hardness.

### 3.3. Phase Transformation

**Figure 6:**
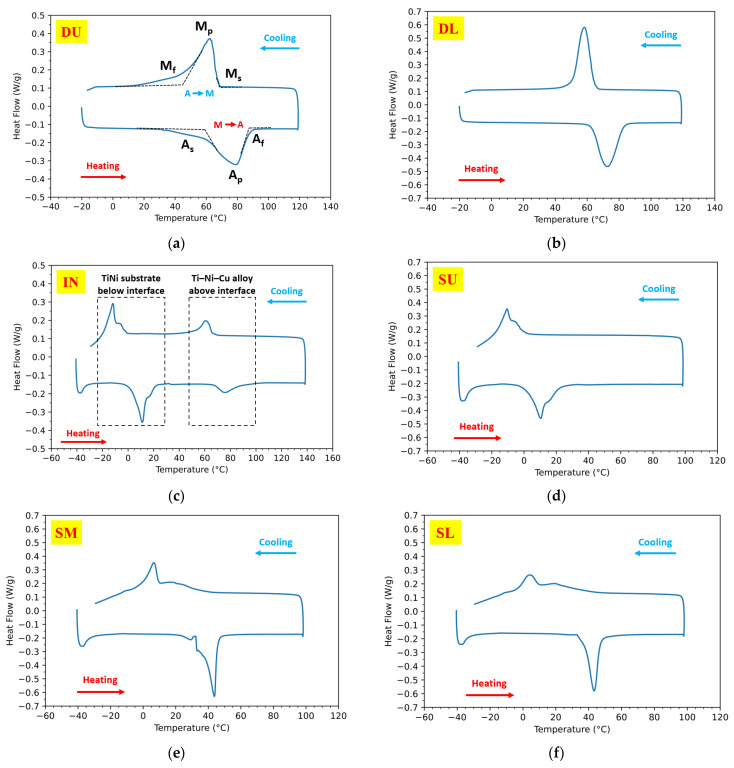
DSC heating–cooling curves of the six regions (marked in Figure 1b) of the as-deposited bi-metallic SMA. (**a**) The upper part of the DED Ti–Ni–Cu alloy (DU). The characterization temperatures: austenite starting temperature (A_s_), austenite peak temperature (A_p_), austenite finishing temperature (A_f_), martensite starting temperature (M_s_), martensite peak temperature (M_p_), and martensite finishing temperature (M_f_) are labeled. (**b**) The lower part of the DED Ti–Ni–Cu alloy (DL). (**c**) The interfacial region (IN). Notice that there are two distinctive phase-transformation peaks during heating and cooling from Ti–Ni–Cu alloy and TiNi substrate, which are marked by dashed boxes. (**d**) The upper part of the substrate (SU). (**e**) The middle part of the substrate (SM). (**f**) The lower part of the substrate (SL).

DSC results in all six different sections: DU, DL, IN, SU, SM, and SL, according to Figure 1b, are demonstrated in Figure 6 from Figure 6a–f, respectively. Each DSC curve consists of a heating and cooling cycle with peaks that indicate the austenite formation during heating and the martensite formation during cooling. Typical characterization temperatures, including martensite starting temperature (M_s_), martensite finishing temperature (M_f_), austenite starting temperature (A_s_), and austenite finishing temperature (A_f_), are determined by the tangent method [32] as shown in the DSC curve of sample DU in Figure 6a. Austenite peak temperature (A_p_) and martensite peak temperature (M_p_) are also included.

Table 1 summarizes the A_f_ of all sections. Figure 6 shows that the Ti-rich upper Ti–Ni–Cu alloy shows a higher austenite finishing temperature A_f_. As for the sections DU and DL, the TiNi matrix is highly Ti-rich and the A_f_ reaches higher than 80 °C. The DSC curve of the IN sample with the interface has two distinctive peaks both during heating and during cooling in Figure 6c, which are marked by dashed boxes. The high-temperature peaks come from the Ti-rich Ti–Ni–Cu alloy portion above the interface, whereas the low-temperature peaks are due to the equiatomic TiNi substrate below the interface. So, after the joining by DED, both phase-transformation behaviors of the two sections near the interface can still be clearly illustrated in DSC. For the substrate, at the upper part of the substrate, which includes the lower half of IN and the section of SU, the values of A_f_ are as low as 21.2 °C and 22.8 °C, whereas the A_f_ values of the SM and SL are 46.5 °C and 47.7 °C.

It can also be seen that all of the samples from the Ti–Ni–Cu alloy section exhibit one-step phase transformation in both the heating and cooling processes. For the substrate, during cooling the upper half of the substrate, IN and SU, exhibit features close to one-step martensite transformation, whereas the cooling curves of the lower part including SM and SL are closer to double overlapped peaks during cooling, which could be related to the intermediate R-phase [33,34] that initially exists in the substrate. For the near-equiatomic TiNi substrate used in this work, the upper part went through repeated high-temperature annealing from the laser, which tends to exhibit lower TTs and one-step austenite-martensite transition after annealing [33,35]. The lower part of the substrate, SM and SL, which are far from the deposited part, underwent a relatively lower heating temperature; the TTs are not highly affected by the laser-annealing processes. It was also reported that the formation of the R-phase can be favored by secondary precipitates such as Ni_4_Ti_3_ and dislocation substructures [36]. Therefore, the higher hardness values of the lower substrate from Z = 1 mm to Z = 5 mm are likely from the existence of precipitates and dislocation substructures. In contrast, the high-temperature annealing effect at the upper substrate reduces the hardness due to the reduction in those factors.

The thermal hysteresis ΔT is defined by the temperature difference between A_p_ and M_p_ [37]. The ΔT value of the DED Ti–Ni–Cu alloy section was calculated for the whole Ti–Ni–Cu alloy section. The ΔT values of DU, DL, and the upper part of IN ranges from 14.4 °C to 17.2 °C, which is narrower than reported additively manufactured Ti-rich TiNi binary SMAs such as in [38]. This then shows the effects of the addition of the Cu element in using the AM process to fabricate TiNi-based SMAs.

### 3.4. Tensile Behavior

**Figure 7:**
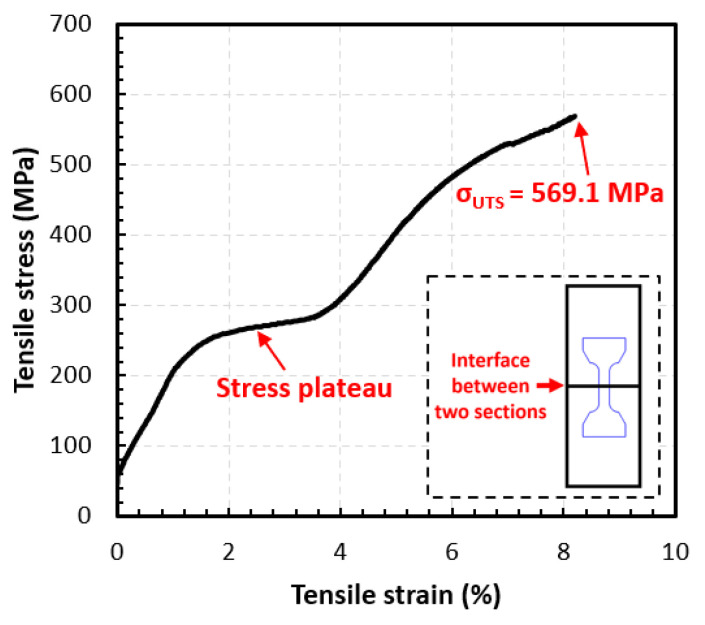
The stress–strain curve of the DED as-deposited bi-metallic structure. The miniature tensile sample was extracted with the interfacial line located at the center of the gauge section.

**Figure 8:**
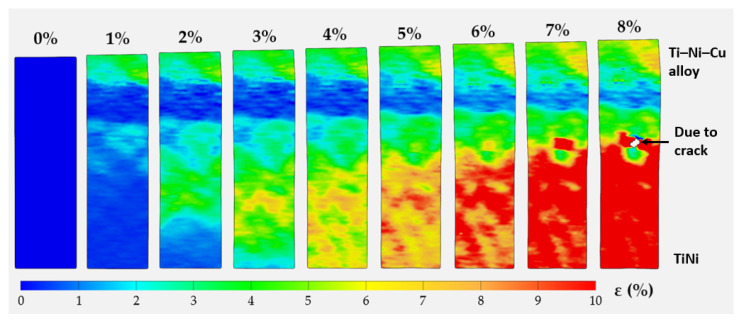
The strain map of the local axial strain within the gauge section at the moments when the total tensile strain equals 0, 1%, 2%, 3%, 4%, 5%, 6%, 7%, and 8%.

The DIC technique was applied using GOM Correlate software to plot the stress–strain curve of the bi-metal under a uniaxial load using the function of the virtual extensometer. A testing sample was extracted vertically across the interface with the half-gauge section belonging to the DED Ti–Ni–Cu alloy single wall and the other half gauge located within the TiNi substrate according to the design in Figure 2a, as seen in the dashed rectangular box in the lower right corner of Figure 7. Thus, the axial direction of the sample and the tensile loading direction are parallel to the Z axis shown in Figure 1. The tensile stress–strain curve is shown in Figure 7, which demonstrates the typical stages of SMA. In order to obtain a good alignment on the miniature tensile specimen, a preload was applied at the beginning of the uniaxial tensile testing. The first near-linear section mainly corresponds to the initial elastic response. After the initial elastic stage, the curve starts a near-horizontal stress plateau, which mainly comes from the stress-induced martensite formation of the austenite-dominated top part of the TiNi substrate (the lower half of the IN section) [39]. After the plateau, the curve goes into the elastic stage of the detwinned martensitic structure, followed by plastic deformation after the elastic region [39]. It can be seen from the stress–strain curve of the sample in Figure 7 that the sample obtains an ultimate tensile strength $\sigma_{UTS}$ of 569.1 MPa. The total tensile strain at the fracture is about 8.2%. The local axial strain (ε) within the gauge section when the total tensile strain reaches 0, 1%, 2%, 3%, 4%, 5%, 6%, 7%, and 8% are mapped and shown in Figure 8. From the entire evolution of the strain map, it can be observed that as the tensile load increases, the gauge shows two distinctive sections with higher local axial strain in the TiNi substrate (approximately 10%) and lower local axial strain in the DED Ti–Ni–Cu alloy (approximately 4~5%). Anomalous points start to appear in the strain map near the interface when the total tensile strain reaches 8% due to crack formation. Therefore, the mechanical behavior at different sections of the bi-metallic SMA can be clearly tracked using the DIC technique.

**Figure 9:**
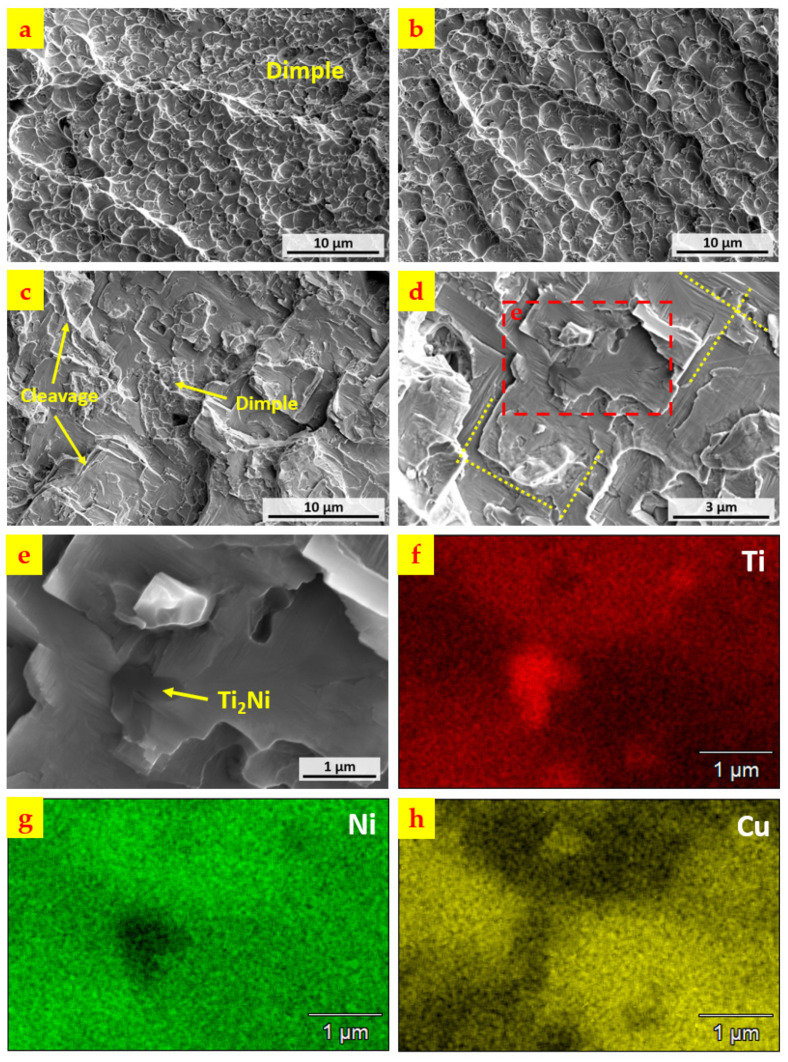
(**a**,**b**) Dimple-like ductile area of the fracture surface. (**c**,**d**) Brittle area of the fracture surface. (**e**) High magnification image of (**d**) in long, dashed rectangular box. (**f**) Element mapping of Ti in (**e**). (**g**) Element mapping of Ni in (**e**). (**h**) Element mapping of Cu in (**e**).

The SEM images of the details on the fracture surface of the sample are shown in Figure 9. Figure 9a,b shows the ductile regions where the dimple-like structure dominates, whereas the feature in Figure 9c,d shows low ductility. Figure 9c includes both local dimples and cleavage regions, and in the higher magnification image of Figure 9d, it also has perpendicular patterns marked by perpendicular short, dashed lines, which may correspond to the martensite twinning structure within the Ti–Ni–Cu alloy single-wall section shown in Figure 3d. Also, the dark particles can be found in Figure 9d. Figure 9e–h represent the SEM/EDS mapping of the long, dashed rectangular area in Figure 9d. The Ti, Ni, Cu element mapping of Figure 9e indicates that the dark particle could be Ti_2_Ni, which may act as a stress concentrator near the interface that initiates the cracking under the tensile loading.

### 3.5. Demonstration of Multiple Shape-Memory Effect

**Figure 10:**
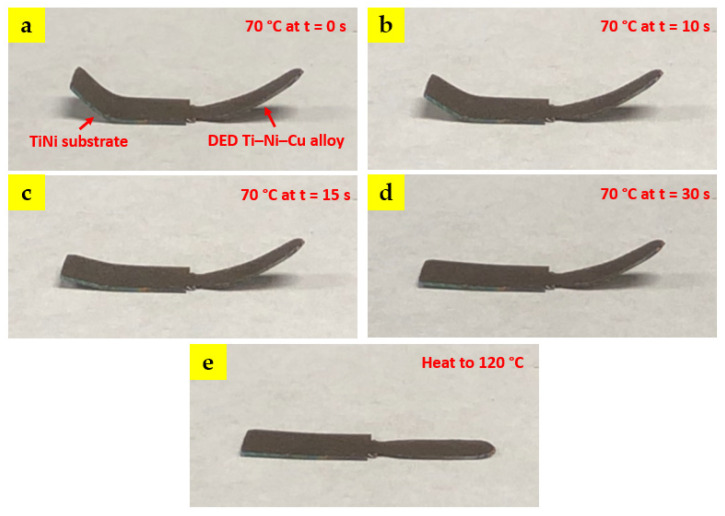
Demonstration of the SMA using a hot plate at 70 °C and 120 °C: (**a**) The bi-metallic SMA sample was bent at both the TiNi substrate side and the DED Ti–Ni–Cu alloy side and placed at 70 °C at t = 0 s. (**b**) t = 10 s. (**c**) t = 15 s. (**d**) t = 30 s. Notice that the TiNi substrate has almost recovered to the original status, whereas the Ti–Ni–Cu alloy side keeps the curved shape. (**e**) The hot plate was then heated from 70 °C to 120 °C. Finally, the Ti–Ni–Cu alloy side recovers.

The multiple shape-memory behavior is demonstrated by a cross-section slice from the as-deposited bi-metallic SMA. Both the DED Ti–Ni–Cu alloy side and the TiNi substrate side are bent compared to the original straight shape. After bending, the bi-metallic SMA piece was then placed on a hot plate at 70 °C, which is higher than (A_f_ + 20 °C) of the TiNi substrate, as seen in Figure 10a. Figure 10b–d capture the shape change of the bi-metallic SMA on the hot plate at 70 °C after 10 s, 15 s, and 30 s, respectively. It can be noticed that at 70 °C, the curved TiNi substrate side gradually changes back to the straight status. In comparison, the Ti–Ni–Cu alloy side keeps the curved shape since the temperature of 70 °C is not able to complete the martensite-austenite transition of the Ti–Ni–Cu alloy section. As the time reaches 60 s, the hot plate temperature starts to rise and finally reaches 120 °C. Figure 10e shows that at 120 °C, which is higher than the (A_f_ + 20 °C) of the DED Ti–Ni–Cu alloy part, the Ti–Ni–Cu alloy side also recovers to the original straight state. Therefore, the multiple shape-memory behaviors at two sides of the bi-metallic structure are identified.

## 4. Conclusions

In this work, a Ti-rich Ti–Ni–Cu ternary SMA with an as-mixed atomic composition of Ti–44at.%Ni–3at.%Cu was fabricated on a near-equiatomic commercial TiNi binary SMA substrate, which shows the capability of the elemental powder DED process to join two different types of SMA sections and build SMAs with multiple shape-memory behaviors. The findings from studying the microstructural, mechanical, and functional behaviors of the as-fabricated bi-metallic SMA are summarized below.

Microscopic imaging revealed the dense metallurgical bonding between the as-deposited Ti–Ni–Cu alloy part and the TiNi substrate with no gas pores or cracks being seen at the interface. The TiNi matrix phase and Ti_2_Ni minor phase are identified by EDS, and the martensite twinning structure is observed within the TiNi matrix phase of the as-deposited Ti–Ni–Cu alloy section.

Vickers hardness distribution from bottom to top shows the typical low hardness (200~300 HV 0.2) of SMAs across the entire cross-section. All major phases are TiNi phases rather than Ti_2_Ni hard intermetallic phases, and Ti_2_Ni is the main secondary phase in the Ti–Ni–Cu alloy section.

Tensile testing combined with the DIC technique on a miniature tensile sample shows a bonding strength of 569.1 MPa and the fracture occurs at 8.2% total tensile strain. DIC strain mapping indicates the difference in local axial strain distribution between two sections of different SMA types, which are approximately 4~5% in the Ti–Ni–Cu alloy side and approximately 10% in the TiNi substrate.

DSC analysis at different sections shows that the Ti-rich DED Ti–Ni–Cu alloy shows higher A_f_. For the near-equiatomic TiNi substrate, the lower half of the substrate maintains a higher A_f_ and more obvious R-phase features than the upper half of the substrate since the upper part went through higher temperature annealing from laser power, which tends to exhibit lower TTs and a single austenite-martensite transition without an R-phase.

The shape-memory behavior is tested by an as-deposited bi-metallic slice at 70 °C and 120 °C. At 70 °C, shape recovery occurs only at the TiNi substrate side, whereas when the temperature reaches 120 °C, both sides perform the shape recovery. Future studies will include the heat treatment effect on the microstructure and functional properties of DED bi-metallic SMAs.

## Figures and Tables

**Table 1:** Summary of the A_f_ value of all sections within the bi-metallic SMA structure and the corresponding Z heights.

Sample Section	A_f_ (°C)
DU	88.3
DL	82.9
IN (above interface)	90.9
IN (below interface)	21.2
SU	22.8
SM	46.5
SL	47.7

## Data Availability

Not applicable.
